# Occurrence and Health Risk Assessment of Per- and Polyfluoroalkyl Substances in Yogurt Across Lebanese Governorates

**DOI:** 10.3390/foods14203472

**Published:** 2025-10-11

**Authors:** Sandra Sarkis, Maha Hoteit, Nikolaos Tzenios, Tony Tannous, Mireille Harmouche-Karaki, Khalil Helou, Joseph Matta

**Affiliations:** 1 Department of Nutrition, Faculty of Pharmacy, Ecole Doctorale Sciences et Santé (EDSS), Medical Sciences Campus, Saint Joseph University of Beirut (USJ), Beirut 1100, Lebanon; 2 PHENOL Research Group (Public Health Nutrition Program-Lebanon), Faculty of Public Health, Lebanese University, Beirut 1100, Lebanon; m.hoteit@ul.edu.lb; 3 Department of Primary Care and Population Health, University of Nicosia Medical School, P.O. Box 24005, Nicosia 1700, Cyprus; 4 Institut National de Santé Publique, d’Epidémiologie Clinique et de Toxicologie-Liban (INSPECT-LB), Beirut 1100, Lebanon; 5 Faculty of Public Health, Charisma University, London EC1V 7QE, UK; 6 Faculty of Arts and Sciences, University of Balamand, Tripoli P.O. Box 100, Lebanon; tony.tannous@balamand.edu.lb; 7 Department of Nutrition, Faculty of Pharmacy, Saint Joseph University of Beirut (USJ), Beirut 1100, Lebanon; mireille.harmouche@usj.edu.lb (M.H.-K.); khalil.helou@usj.edu.lb (K.H.); joseph.matta@usj.edu.lb (J.M.); 8 Industrial Research Institute, Lebanese University Campus, Hadat Baabda, Beirut 1100, Lebanon

**Keywords:** PFAS, yogurt, food contamination, Lebanon, risk assessment

## Abstract

Per- and polyfluoroalkyl substances (PFAS) are persistent environmental pollutants of emerging concern due to their widespread use and potential adverse health effects. This study assessed the concentrations of key PFAS compounds in yogurt samples collected from eleven Lebanese governorates. Results revealed notable geographic variability, with the Bekaa region exhibiting the highest PFAS levels, particularly PFHpA, PFOA, PFHxS, PFOS, and PFPeA, while Jbeil showed the lowest concentrations. Health risk assessment using estimated daily intake (EDI), risk quotients (RQ), and cumulative hazard index (HI) indicated all individual compound exposures below established safety thresholds. However, elevated RQs for PFOS and PFOA and an average HI of 0.71 suggest potential chronic exposure concerns in high-burden regions. These findings emphasize the importance of continued monitoring and risk management to protect public health and inform environmental policies addressing PFAS contamination in Lebanon.

## 1. Introduction

Per- and polyfluoroalkyl substances (PFAS) are a large group of man-made chemicals widely used for their oil-, water-, and heat-resistant properties in numerous industrial processes and consumer goods, including non-stick cookware, stain-resistant fabrics, food packaging, and firefighting foams [1,2]. Over the past few decades, PFASs have garnered global attention due to their persistence in the environment, ability to bioaccumulate in living organisms, and growing evidence of adverse effects on human health, including developmental, immunological, endocrine, and carcinogenic outcomes [1,2]. These properties have earned PFAS the moniker “forever chemicals,” as they resist degradation and tend to remain in ecosystems and the human body for extended periods [3].

While initial research on PFAS contamination centered largely around drinking water and occupational exposure, emerging findings reveal that food can be a significant pathway for human exposure. This includes contamination through food processing, packaging, and the bioaccumulation of PFAS in agricultural and animal-derived products [3]. Dairy products are increasingly being scrutinized due to the potential transfer of PFAS from contaminated water, soil, or feed into milk and its derivatives.

Lebanon, an eastern Mediterranean country, is divided into four main zones from west to east: a coastal plain, the Mount Lebanon mountain range, the fertile Bekaa Valley, and the Anti-Lebanon mountains bordering Syria [4]. Within this context, dairy, especially artisanal yogurt, traditionally produced and commonly consumed in Lebanon, is an integral component of the traditional diet, consumed daily across socio-economic and age groups. Yogurt holds a prominent place in Lebanese cuisine due to its health benefits, cultural value, and culinary versatility, making it a potential vector for PFAS dietary exposure. The Lebanese dairy industry has witnessed steady growth despite economic and political challenges, with yogurt production and consumption remaining central to national food habits [5]. However, amid growing global concern over environmental pollutants, Lebanon still lacks comprehensive surveillance of PFAS contamination in food and water sources. The absence of national guidelines, standardized testing protocols, and spatially representative data on PFAS levels presents a major challenge for health authorities aiming to safeguard public health.

Furthermore, Lebanon’s fragile environmental infrastructure, compounded by inadequate waste management systems and unregulated industrial discharge, may increase the risk of PFAS release into ecosystems. This environmental vulnerability raises the possibility of PFAS infiltrating the food chain, yet the extent and distribution of such contamination remain largely unquantified.

Addressing this critical gap, the present study investigates the presence and distribution of PFAS in yogurt samples collected from eleven governorates across Lebanon. The study specifically aims to:

- Quantify the concentrations of key PFAS compounds, including PFHpA, PFOA, PFNA, PFDA, PFHxS, PFOS, PFHxA, PFBA, PFBS and PFPeA, in yogurt products sourced from diverse regions;

1. Assess the spatial variation of PFAS contamination to identify potential environmental hotspots that may be contributing to regional differences in exposure;
2. Evaluate health risks associated with PFAS ingestion through yogurt consumption by calculating the estimated daily intake (EDI), risk quotients (RQ), and cumulative hazard indices (HI);
3. Inform public health and environmental management by generating region-specific scientific evidence to support the development of national monitoring frameworks and guide future policy interventions on PFAS in Lebanon.

## 2. Materials and Methods

### 2.1. Sampling

#### 2.1.1. Sample Collection and Regional Context

This study involved the collection of yogurt samples from eleven distinct regions across Lebanon, including Nabatieh (Na), South Lebanon (S), Chouf (C), Metn (M), Keserwan (K), Jbeil (J), Batroun (B), North Lebanon (No), Akkar (A), Bekaa (Be), and Baalbeck-Hermel (BH). The sampled Lebanese regions reflect diverse agro-economic and environmental conditions. Southern areas (Nabatieh, South Lebanon) rely heavily on agriculture (tobacco, olives) but face pressures from urban expansion, industrial activity, poor waste disposal, and pesticide runoff [6]. Mountainous districts (Chouf, Metn, Keserwan) combine farming with urban or industrial growth, with challenges including agrochemical use, vehicle emissions, and wastewater discharge [7]. Coastal regions (Jbeil, Batroun, Lebanon) balance agriculture, fishing, and tourism but suffer from unmanaged waste, and marine pollution [7].

Northern rural zones (Akkar, North Lebanon) depend on farming yet struggle with banned agrochemicals, pesticides residues, untreated discharges, and weak waste management [8,9]. The Bekaa Valley and Baalbeck-Hermel are agricultural heartlands but face soil and water pollution due to fertilizer overuse and agricultural runoff, industrial pollution, and inadequate waste treatment [10,11,12].

Table S1 shows the Central GPS coordinates of yogurt sampling locations across Lebanese governorates of each region. From each region, yogurt samples were collected by trained staff from the Industrial Research Institute (the Industrial Research Institute is accredited by ANAB, USA for ISO 17025 (testing and calibration) and ISO 17020 (Inspection) and member of the International Accreditation Forum (IAF) and International Laboratory Accreditation Cooperation (ILAC), AND European Federation of National Associations of measurement, testing and analytical Laboratories (EUROLAB)) (IRI). From each farm, one yogurt sample (500 g) was obtained, resulting in 15 independent samples per governorate and a total of 165 samples across the country. Sampling was conducted following an accredited procedure adopted by IRI and based on the Codex Alimentarius General Guidelines on Sampling [13]. All samples were collected in a standardized manner, under sterile conditions using sterile gloves and aseptic transfer, ensuring standardization and minimizing the risk of external contamination

**Figure 1:**
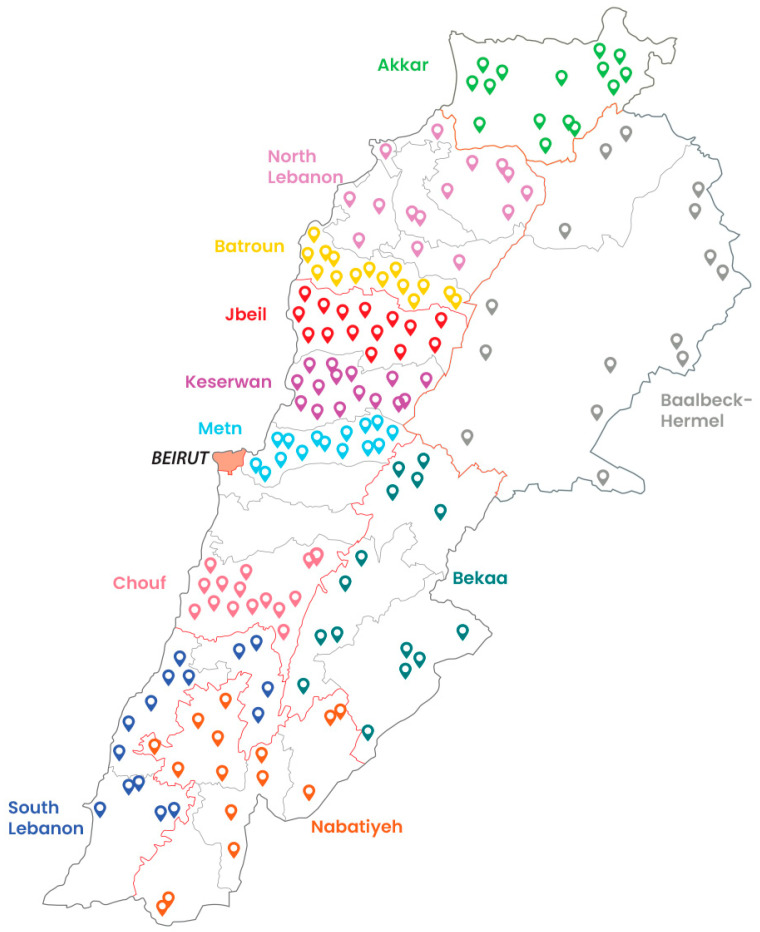
Geographical distribution of sampling locations across the 11 governorates.

All collected yogurts were traditionally produced on-site using raw cow’s milk sourced locally, without the addition of preservatives, stabilizers, or artificial flavors, and they do not represent commercial brands. Fermentation was carried out with natural starter cultures obtained from previous yogurt batches (“back-slopping”), rather than commercial cultures, and relied mainly on the naturally occurring lactic acid bacteria most commonly, Lactobacillus delbrueckii subsp. bulgaricus and Streptococcus thermophilus, which are characteristic of traditional Lebanese yogurt. All yogurt samples were stored in food-grade polypropylene containers, under sterile conditions using sterile gloves and aseptic transfer, ensuring standardization and minimizing the risk of external contamination. All samples were collected in the same manner during the spring of 2025 (March–May), when ambient temperatures averaged 18 °C. Samples were transported in portable coolers with ice packs at 4 °C. Given Lebanon’s small geographic size, with the longest distance between farms and the laboratory being less than 100 km, transport was completed quickly to minimize any potential changes in sample integrity. Upon arrival at the Industrial Research Institute laboratories, samples were immediately refrigerated (4 °C) and subjected to chemical analysis without delay to accurately determine the presence and concentration of per- and polyfluoroalkyl substances (PFAS). The geographical distribution of sampling locations is illustrated in Figure 1.

#### 2.1.2. Environmental and Socioeconomic Overview of Each Sampled Region Regarding PFAS

Recent evidence highlights the presence of PFAS in several Lebanese environmental compartments, though data remain fragmented and region-specific comparisons are scarce. The Lebanon PFAS Country Situation Report indicate that PFAS sources in Lebanon include firefighting foams, industrial uses, food packaging, wastewater, and landfill leachates, but systematic monitoring across governorates is lacking [14]. Peer-reviewed biomonitoring studies confirm measurable PFAS levels in Lebanese populations: maternal serum, cord blood, and human milk all showed detectable concentrations, demonstrating prenatal and early-life exposure, although these studies reported national-level rather than regionally stratified results [15,16]. Environmental research points to clear hotspots along the coast. Sediment analyses conducted at multiple sites along the Lebanese shoreline revealed persistent organic pollutants, including PFAS, with elevated levels near urban, industrial, and river outlets, underscoring the role of riverine transport in mobilizing inland contamination [9,12,17]. This pathway is consistent with findings on the Litani River in the Bekaa Valley, where agrochemical runoff, industrial effluents, and untreated wastewater are discharged, ultimately carrying contaminants into the Mediterranean [17]. By contrast, rural and inland governorates such as Akkar or Baalbeck–Hermel, although heavily dependent on agriculture, lack direct PFAS measurement studies; instead, evidence of lingering organochlorine pesticide residues and weak waste management suggests that PFAS burdens remain an under-investigated risk in these areas.

### 2.2. Extraction and Instrumental Analysis of PFAS in Yogurt Samples

This study measured ten PFAS compounds: PFHpA, PFOA, PFNA, PFDA, PFHxS, PFOS, PFHxA, PFBA, PFBS, and PFPeA in yogurt samples

#### 2.2.1. Reagents and Materials

The PFAS analytical standards, with a chemical purity of >98% for each compound, were purchased from Wellington Laboratories (Guelph, ON, Canada). HPLC-grade methanol (MeOH), acetonitrile (MeCN), and water were obtained from Fisher Scientific (Fair Lawn, NJ, USA). Formic acid (≥99%) was supplied by Merck (Darmstadt, Germany), and glacial acetic acid was purchased from VWR International (Leuven, Belgium). Anhydrous magnesium sulfate p.a. (MgSO_4_) and sodium chloride p.a. (NaCl) were obtained from POCh SA (Gliwice, Poland). The ENV (styrene–divinylbenzene) SPE bulk sorbent was supplied by Agilent Technologies (Santa Clara, CA, USA), and water was purified using a Milli-Q system (Millipore, Bedford, MA, USA).

Standard stock solutions (10 µg/mL in methanol) were diluted in 20% MeOH (*v*/*v*) containing 1% formic acid to prepare calibration standards ranging from 0.05 to 50 ng/mL. Internal standards were spiked at 100 ng/mL into each yogurt sample prior to extraction to ensure accuracy of quantification.

#### 2.2.2. Sample Extraction and Cleanup

In this study, concentrations of PFASs in yogurt samples were determined using the QuEChERS method with a dispersive SPE (d-SPE) clean-up step as described by Sznajder-Katarzyńska et al. [18] prior to HPLC-MS/MS analysis. Specifically, 10 g of homogenized yogurt was weighed into a 25 mL polypropylene centrifuge tube and spiked with 10 µL of an internal standard solution (100 ng/mL) [18]. Extraction was performed with 10 mL MeCN and 150 µL formic acid (FA) by sonication (2.5 min, 40 kHz) followed by mechanical agitation for 1 min. Subsequently, 1 g NaCl and 4 g MgSO_4_ were added, and the mixture was shaken for 1 min. Samples were centrifuged for 15 min at 8693× *g* at 4 °C to achieve phase separation. Subsequently, 1 g NaCl and 4 g MgSO_4_ were added, and the mixture was shaken for 1 min to induce a salting-out effect and to remove residual water, thereby improving phase separation [18].

From the separated acetonitrile layer, 6 mL were transferred into a 15 mL polypropylene tube containing 0.15 g ENV SPE bulk sorbent and 0.90 g MgSO_4_ for dispersive clean-up [18]. Tubes were shaken for 30 s, centrifuged for 5 min at 8693× *g*, and 4 mL of the supernatant were collected into a 4 mL tube. The extracts were then stored at −12 °C for 20 h to precipitate residual fat and subsequently filtered through Whatman No. 1 filter paper [18]. After filtration, the filtrates were evaporated to dryness in a vacuum concentrator (40 °C, ~2 h). The residues were reconstituted in 1 mL MeOH and diluted fivefold with ultrapure water containing 1% (*v*/*v*) formic acid before HPLC-MS/MS analysis.

All extractions were performed in triplicate, and method blanks and reagent blanks were included in each batch to monitor background contamination and ensure reproducibility.

#### 2.2.3. Instrumental Analysis

Instrumental conditions were similar to those described by Mahfouz et al. [15]. A Vanquish extractor (Thermo Fisher, Waltham, MA, USA) equipped with an autosampler and HPLC pump was operated using the Chromeleon software (Thermo Fisher, Waltham, MA, USA). The HPLC system was configured with a 1000 µL/min flow rate, using 95% of 20 mM ammonium acetate (pH 4) and 5% acetonitrile as mobile phase A, and 100% acetonitrile as mobile phase B. Analytes were separated on two Chromolith^®^ HighResolution RP-18e columns (4.6 × 100 mm) preceded by a guard column (5 × 4.6 mm) and a Chromolith^®^ HighResolution RP-18e column (4.6 × 25 mm) or equivalent. To delay elution of PFAS contaminants from Teflon pump components, an additional 4.6 × 25 mm Chromolith^®^ HighResolution RP-18e column was inserted between the HPLC pump and the right clamp valve.

Detection was performed using an Orbitrap Exploris or a TSQ Quantis Plus triple quadrupole mass spectrometer (Thermo Fisher) operated in negative electrospray ionization (ESI) mode, with a TIS ionization source for converting liquid-phase ions into gas-phase ions. Detection was carried out in selected reaction monitoring (SRM) mode with optimized precursor-to-product ion transitions for each PFAS analyte. Calibration was achieved using matrix-matched external standards (0.05–50 ng/mL), which showed linearity with R^2^ ≥ 0.99.

Quality Assurance/Quality Control (QA/QC): Limits of Detection (LOD) ranged from 0.05 to 0.1 ng/mL depending on analyte, with Limits of Quantification (LOQ) set at three times the LOD. Method recoveries ranged from 80 to 120% across three spiking levels, and precision expressed as relative standard deviation (RSD) was ≤12%.

### 2.3. Quality Control and Assurance

For each batch, analytes were quantified using individual calibration curves, which showed linearity across the tested range (regression coefficients: 0.98–0.99). The upper limit of linearity corresponded to the highest standard analyzed, while concentrations below the method LOD or the lowest calibration standard were reported as not detected.

The limits of detection (LOD) and corresponding quantification ranges were as follows: PFOA, 0.1 ng/mL; PFOS, 0.1 ng/mL; PFHpA, PFHxS, PFDA, PFNA, PFHxA, PFBA, PFBS and PFPeA 0.05 ng/mL. Limits of quantification (LOQs) were estimated as three times the LOD values: PFOA, 0.3 ng/mL; PFOS, 0.3 ng/mL; PFHpA, PFHxS, PFDA, PFNA, PFHxA, PFBA, PFBS and PFPeA 0.15 ng/mL [19].

Method accuracy was evaluated by spiking serum samples with known PFAS concentrations at three different levels between 3× LOD and 30 ng/L, yielding recoveries of 80–120%. Precision, expressed as total relative standard deviation, was ≤12% [20].

### 2.4. Data Analysis

Data analysis was performed using IBM SPSS Statistics (version 26). Descriptive statistics, including means and standard deviations, were used to summarize and compare the concentrations of PFAS detected in yogurt samples across different Lebanese regions. To assess compliance with regulatory standards, one-sample two-tailed *t*-tests were applied to compare the observed contaminant levels with the established maximum residue limits (MRLs).

### 2.5. Methodology: Risk Assessment

To evaluate the potential health risks associated with dietary exposure to per- and polyfluoroalkyl substances (PFASs) through yogurt consumption in Lebanon, a quantitative risk assessment approach was adopted, integrating the calculation of Estimated Daily Intake (EDI), Risk Quotient (RQ), and Hazard Index (HI). PFAS concentrations (expressed in ng/kg) in yogurt samples were first measured and used to estimate the daily intake of each compound based on the following formula: EDI (ng/kg bw/day) = (Concentration × Daily Intake)/Body Weight. Risk assessment was restricted to adults because validated national data on yogurt consumption and body weight were available for this subgroup through the LEBANON-FCS survey [21]. Comparable data for children and adolescents were not accessible at the time of analysis, limiting the feasibility of accurate exposure estimation for younger populations. In this calculation, the average daily yogurt consumption among Lebanese adults was set at 72.3 g (0.0723 kg/day) [21], and the mean adult body weight was assumed to be 73.85 kg [21]. Subsequently, the RQ for each individual PFAS was determined by dividing the EDI by the compound’s respective toxicity reference value—either a Reference Dose (RfD) or a Tolerable Daily Intake (TDI), according to the equation: RQ = EDI/(RfD or TDI). Values for RfDs and TDIs were sourced from internationally recognized authorities, including the United States Environmental Protection Agency (USEPA) and the European Food Safety Authority (EFSA). For example, the RfD for PFOA was set at 1.5 ng/kg bw/day [22] and for PFOS at 1.8 ng/kg bw/day [23]. In cases where official reference doses were unavailable, surrogate values or Relative Potency Factors (RPFs) derived from recent literature were applied to maintain consistency [24] (Refer to Table 1). To evaluate the combined risk from multiple PFAS compounds, the Hazard Index (HI) was calculated as the sum of the individual RQs across all detected substances: HI = ∑ RQᵢ, where RQᵢ represents the risk quotient of each compound and n is the number of detected PFAS. An HI value exceeding 1 is considered indicative of potential health concern due to the additive or synergistic effects of PFAS mixtures [1,22]. Where applicable, PFOA Equivalent Doses (PEQs) were also computed based on established RPFs to allow for cumulative toxicity assessment on a standardized toxicological scale, thereby aiding in prioritizing PFAS according to their relative hazard.

## 3. Results

### 3.1. PFA Concentrations Across the Lebanese Governorates

**Figure 2:**
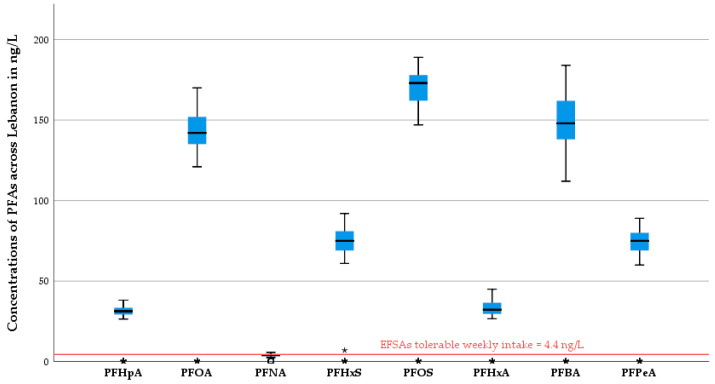
Boxplots of the Concentrations of the various perfluoroalkyl carboxylic acid (PFAs) in Lebanon. *: extremely outlying value where the difference from other values is highly significant and it falls much farther away from the whiskers of the plot.

The analysis of PFAS concentrations across Lebanese governorates revealed notable geographic variation in exposure levels. The Bekaa region exhibited the highest concentrations for multiple PFAS compounds, particularly PFHpA, PFOA, PFHxS, PFOS, and PFPeA, indicating a potential hotspot of environmental contamination. North Lebanon and Beirut-Hermel (BH) also showed relatively elevated concentrations across several compounds, especially PFOS and PFOA. In contrast, Jbeil consistently recorded the lowest levels across most PFAS, including PFNA, PFHxS, and PFOS. The average concentrations across all governorates for each compound were as follows: PFHpA (32.01 ng/L), PFOA (145.41 ng/L), PFNA (4.09 ng/L), PFHxS (75.62 ng/L), PFOS (172.12 ng/L), PFHxA (33.67 ng/L), PFBA (150.54 ng/L), and PFPeA (75.44 ng/L). PFDA and PFBS were not detected in any of the samples, suggesting either their absence or concentrations below detection limits (Table 2 and Figure 2).

### 3.2. Comparison Between Lebanese Governorates

A one-sample, two-sided t-test was conducted to assess the concentrations of eight Perfluoroalkyl Carboxylic Acids (PFAs) across 11 Lebanese regions. PFDA and PFBS were excluded from the analysis as their concentrations were below the limit of detection (LOD) in all samples. For all PFAs except PFNA, concentrations were substantially above the intake limit of 4.4 ng/L. PFNA levels were closer to the limit, with variability across regions. PFHpA concentrations were significantly elevated in all regions (*p* < 0.001), with 95% confidence intervals (CIs) ranging from 17.42 to 37.86 ng/L. The lowest mean range was observed in Jbeil (17.42–31.38 ng/L) and the highest in Bekaa (27.67–37.86 ng/L). PFOA levels were consistently high across regions (*p* < 0.001), with CIs spanning from 76.68 to 159.4 ng/L. Bekaa (147.8–159.4 ng/L) and Baalbeck-Hermel (145.4–155.4 ng/L) exhibited the highest means. PFNA concentrations showed regional variability, with significant differences in some areas (e.g., Baalbeck-Hermel: 4.576–5.091 ng/L, *p* = 0.003) and non-significant results in others (e.g., Bekaa: 3.666–5.041 ng/L, *p* = 0.886). Only 33.9% (56/165) of PFNA samples exceeded the intake limit. PFHxS concentrations were significantly elevated in all regions (*p* < 0.001), ranging between 39.62 and 84.38 ng/L. The highest values were recorded in South Lebanon (52.77–84.38 ng/L) and Nabatiyeh (51.76–82.05 ng/L). PFOS levels were among the highest measured, with all regions significantly exceeding intake limits (*p* < 0.001) and CIs ranging from 114.3 to 188.3 ng/L. Bekaa (119.0–188.3 ng/L) and Baalbeck-Hermel (137.3–187.9 ng/L) showed particularly elevated concentrations. PFHxA levels were also significantly above the intake limit across regions (*p* < 0.001), ranging from 18.29 to 40.82 ng/L. Bekaa (37.23–40.64 ng/L) and North Area (25.70–40.82 ng/L) had the highest ranges. PFBA concentrations were high in all regions (*p* < 0.001), spanning from 96.51 to 164.6 ng/L. Bekaa (148.6–164.6 ng/L) and Baalbeck-Hermel (152.2–163.1 ng/L) were the most contaminated. PFPeA was also consistently elevated (*p* < 0.001), with CIs between 40.85 and 83.32 ng/L. The highest values were recorded in South Lebanon (51.96–83.32 ng/L) and Bekaa (73.36–80.91 ng/L) (Table 2). A one-way ANOVA revealed significant regional differences only for PFNA (F = 4.754, *p* < 0.001) and PFHxA (F = 3.047, *p* = 0.001). No statistically significant differences were found across regions for PFHpA, PFOA, PFHxS, PFOS, PFBA, or PFPeA (Table 3).

### 3.3. Health Related Risk Assessment

#### 3.3.1. Calculation of Estimated Daily Intake and Risk Quotient

In terms of risk assessment, the estimated daily intake (EDI) values reflected a similar regional trend to the raw concentrations, with the highest EDIs observed in the Bekaa, North Lebanon, and BH, particularly for PFOS (0.1735 µg/kg/day in Bekaa) and PFOA (0.1504 µg/kg/day in Bekaa). The EDI values for all compounds remained below established tolerable intake thresholds yet showed a cumulative burden in certain governorates. The risk quotient (RQ), which compares EDI to reference dose (RfD), was consistently below 1 for all PFAS across all regions, indicating no immediate non-carcinogenic risk. However, elevated RQs were noted for PFOS (average RQ: 0.2675; Bekaa: 0.2754) and PFOA (average RQ: 0.2260; Bekaa: 0.2387), approaching levels of potential concern in several governorates. These findings suggest that while current exposure may not pose acute health risks, chronic exposure—especially in high-burden areas like Bekaa and North Lebanon—warrants further investigation and monitoring. PFNA, PFHxA, PFBA, and PFPeA showed very low RQs (ranging from 10^−5^ to 10^−2^), reflecting minimal risk under current exposure levels (Table 4).

#### 3.3.2. Calculation of the Hazard Index (HI)

The average Hazard Index (HI) across all measured locations is approximately 0.71, which is below the threshold value of 1 that typically indicates potential health concern. An HI less than 1 suggests that the combined exposure to the group of PFAS compounds at these locations is generally within acceptable risk levels, implying no immediate cumulative health risk based on current reference doses. Individual site HIs range from about 0.64 to 0.71, consistently remaining under this safety benchmark. However, since the values are relatively close to 1, ongoing monitoring and cautious risk management may be advisable to protect sensitive populations and account for possible additional exposure sources (Table 4).

## 4. Discussion

### 4.1. Geographical Variability and Potential Sources of PFAS Contamination

This study represents one of the first investigations in Lebanon to examine PFAS contamination in yogurt, a widely consumed dairy product with high nutritional and cultural value in the Lebanese diet. Given yogurt’s frequent intake across all age groups, the detection of PFAS compounds in samples collected from eleven governorates raises significant public health considerations.

The results revealed the presence of multiple PFAS analytes, notably PFHpA, PFOA, PFHxS, PFOS, PFHxA, PFBA, and PFPeA, in varying concentrations across regions. The Bekaa governorate exhibited the highest concentrations of PFOS and PFOA, consistent with previous reports of intense agricultural and industrial activity in the region, which may contribute to PFAS contamination in the dairy production chain [17]. High levels in North Lebanon and Baalbeck-Hermel (BH) may similarly reflect environmental hotspots influenced by water quality, livestock feeding practices, and contaminated local ecosystems. These findings are consistent with PFAS behavior as persistent environmental pollutants with a strong potential for bioaccumulation through food chains [3,28,29]. In contrast, Jbeil and Batroun recorded the lowest concentrations across most PFAS analytes, suggesting a reduced contamination burden possibly due to cleaner environmental inputs or more controlled agricultural practices.

In addition to agricultural and environmental pathways, several other sources may explain the regional variability of PFAS concentrations observed in this study. Open burning of municipal solid waste (MSW), a widespread practice in Lebanon, has been previously linked to PFAS release into the environment [15]. National reports indicate that MSW is predominantly managed through landfilling (51%) and open dumping (32%), with only 17% recycled or composted [30]. The Lebanon PFAS Country Situation Report identified the landfills of Zahle (Bekaa) and Tripoli (North) as potentially contaminated sites, which is consistent with the elevated PFAS concentrations detected in these governorates [14]. Beyond incineration, PFAS have also been documented in landfill leachates worldwide, despite attempts to remove or isolate PFAS-containing wastes [31,32]. Such leachates, together with industrial and municipal wastewater effluents [31,33], can infiltrate soil and groundwater, ultimately transferring contamination into food crops and livestock [34]. In Lebanon, all categories of bulky waste, including carpets, furniture, and construction debris, are disposed of alongside MSW in landfills, open dumps, or controlled dumps, further increasing the potential for PFAS release [14].

Industrial sources also represent a major contributor to regional contamination. A national survey reported that seven facilities exhibited a high potential for PFAS emissions, three of which were located in the Bekaa (Al Arz Textile Factory, Maliban Glass Factory, Dalal Steel) and three in the South (Ghabris Detergent Factory, Saffieddine Plasti-med, Fine Tissue Factory), in addition to one in Mount Lebanon (Lebanon Co. for Carton Mince & Industry) [14]. The presence of these industries in Bekaa and South Lebanon may therefore explain the elevated PFAS levels observed in these regions. Another important but often overlooked source is firefighting foams, which remain widely used in Lebanon. It was estimated that between 2004 and 2014, approximately 56–167 kg of PFOA or PFOS were released nationally through the use of imported foams [14]. The Lebanese Army continues to rely on older firefighting equipment and flame-retardant outfits, which may also contain PFOS, echoing findings from other countries where military use was a major contamination source innovation development association. Finally, frequent fire accidents at industrial and storage facilities in Lebanon are considered potential hotspots for the release of persistent organic pollutants, including PFAS [14].

In contrast, Jbeil and Batroun recorded the lowest concentrations across most PFAS analytes, suggesting a reduced contamination burden possibly due to cleaner environmental inputs, fewer industrial activities, and limited sources of PFAS release compared with more urbanized or industrialized governorates. These regions also have less intensive waste generation and disposal sites, which may reduce the risk of PFAS entering soil and water systems. Furthermore, local dairy farming in Jbeil and Batroun often relies on smaller-scale, traditional practices, with reduced dependence on industrial feed or packaging, which could further contribute to the lower contamination levels observed.

Finally, the non-detection of PFDA and PFBS across all samples may indicate either their absence in the food production environment or levels below detection thresholds. Overall, this geographic variability underscores the importance of identifying local sources and production conditions that influence PFAS levels in Lebanese dairy.

### 4.2. Comparison with Human Biomonitoring in Lebanon

Although no previous studies have assessed PFAS in yogurt in Lebanon, recent human biomonitoring offers indirect support for food as a major exposure pathway. Hassan et al. (2023) reported detectable levels of PFOS and PFOA in over 80% of breast milk samples from Lebanese mothers, with median PFOA levels (147 pg/mL) exceeding the EFSA’s safety threshold (60 pg/mL) [16]. The study identified significant associations between PFAS levels in breast milk and consumption of cereals, bread, meat, poultry, and dairy products, including yogurt. These findings reinforce the hypothesis that dietary exposure, particularly through dairy, may represent a key PFAS pathway in the Lebanese population [16].

### 4.3. Health Risk Assessment

The Estimated Daily Intake (EDI) results mirrored regional PFAS concentration trends, with Bekaa, North Lebanon, and BH recording the highest intake values—especially for PFOS (Bekaa: 0.1735 µg/kg/day) and PFOA (Bekaa: 0.1504 µg/kg/day). While these values remain below international tolerable intake thresholds, they suggest an elevated exposure burden for individuals regularly consuming yogurt from these regions.

The Risk Quotients (RQ) for all PFAS remained below 1, indicating no immediate non-carcinogenic risk. However, the highest RQs were observed for PFOS (average: 0.2675; Bekaa: 0.2754) and PFOA (average: 0.2260; Bekaa: 0.2387), raising concerns about chronic exposure, especially among vulnerable populations. These findings align with global evidence identifying PFOS and PFOA as the most hazardous PFAS due to their bioaccumulation, persistence, and association with endocrine disruption, immunotoxicity, and developmental toxicity [35].

The Hazard Index (HI), which integrates cumulative non-carcinogenic risk from multiple PFAS compounds, ranged from 0.64 to 0.71, with an overall average of 0.71.Although the Hazard Index (HI = 0.71) and Risk Quotients (RQ < 1) indicate exposures are below acute risk thresholds, they suggest a potential concern for chronic and cumulative exposure, particularly among sensitive populations such as infants, children, and pregnant women, due to the bioaccumulative nature of PFAS and simultaneous exposure through multiple dietary and environmental sources. This is further supported by breast milk data in Lebanon, where PFOA exceeded EFSA thresholds, pointing to potential early-life exposure risks [36]. Although the calculated risk quotients and hazard index values suggest no immediate concern for adults, it is important to recognize that humans are simultaneously exposed to mixtures of PFAS and other contaminants through food. Mixture toxicity, particularly during early developmental stages such as childhood, may lead to health risks even when individual exposures remain below safety thresholds.

As noted by Martínez-Esquivel et al. (2022), early-life exposure to environmental contaminants such as PFAS can interfere with metabolic programming and endocrine regulation, increasing the risk of obesity, insulin resistance, and other chronic conditions later in life [37]. Given their higher food intake relative to body weight and greater sensitivity during critical windows of development, children may face disproportionate risks from PFAS exposure through diet, including dairy products. Future studies should therefore prioritize exposure assessment in children to better inform risk management and protective public health policies.

### 4.4. Context of PFAS in Food in Lebanon

Despite global concern, Lebanon remains at an early stage of PFAS research and has no national surveillance program targeting PFAS in food. To date, no peer-reviewed studies have directly assessed PFAS contamination in yogurt, milk, or other dairy products. The current study fills this critical gap by offering the first empirical data on PFAS exposure through yogurt, one of the most consumed food items in the Lebanese diet.

Environmental studies have indirectly highlighted several pathways through which PFAS may contaminate food in Lebanon. For instance, Hassoun et al. (2021) detected PFAS in surface waters, particularly in areas near urban and agricultural zones, raising concerns about the potential use of contaminated water in livestock and dairy production [38]. Similarly, reports by UNEP and GEF (2019–2022), developed under the framework of the Stockholm Convention, acknowledged the lack of PFAS monitoring in the Lebanese food sector and identified firefighting foams, industrial discharges, and imported goods as possible contamination sources [39]. A broader assessment by Mroueh et al. (2022) emphasized the need to integrate PFAS surveillance into national food safety programs, recognizing Lebanon’s vulnerability to environmental and food chain contamination [40]. The EMERGE Project (2020) further underscored the country’s limited laboratory capacity and recommended expanding monitoring efforts from environmental matrices to include food products [41]. These national and regional findings are consistent with international literature, which confirms that PFAS can enter dairy and other food items through contaminated water, animal feed, or food packaging, reinforcing the importance and timeliness of the current study’s focus on yogurt as a potential source of dietary exposure in Lebanon [3,41].

### 4.5. Regional Comparisons Regarding PFAS

For cross-study comparison, we focused on PFOA and PFOS, the predominant compounds in our dataset, with mean concentrations of 0.132 µg/kg for PFOA and 0.156 µg/kg for PFOS. When compared with European studies, our results were consistently higher. Draghi et al. reported 11.54 pg/g PFOA and 21.64 pg/g PFOS in Italian milk [42], Barbarossa et al. found PFOA only in organic milk and cream, with a range concentration of 0–32 and 0–27 pg mL^−1^, respectively. Conversely, PFOS was found in all of the sample types, in particular with a concentration range of 0–67 pg mL^−1^ in raw milk, 0–26 pg mL^−1^ in skimmed milk and 0–32 pg mL^−1^ in milk cream [43], while Still et al. documented even lower levels in German raw milk of 6.2 pg/g PFOA and 18.9 pg/g PFOS in cream milk [44]. In contrast, a recent Chinese study by Liu et al. reported higher values: 0.5 ng/g PFOA and 0.7 ng/g PFOS [45]. Taken together, our results fall above European values but below the recent Chinese finding, reflecting both environmental burdens and regulatory contexts. In fact, Europe and the United States have applied strict restrictions on long-chain PFASs, particularly PFOS and PFOA, replacing them with short-chain alternatives such as PFBA [46,47,48]. Conversely, China has maintained, and in some cases increased, the production and use of long-chain PFASs, which may explain the higher contamination levels observed in Chinese dairy products [49].

### 4.6. Implications and Recommendations

There is growing evidence that PFAS contamination in food products can be influenced by multiple factors along the production and storage chain. For instance, PFAS quantitation has been shown to be sensitive to storage containers and sample preparation conditions, with losses of up to 50% of certain PFAS reported depending on container material, solvent composition, and storage duration [50]. Similarly, studies of fluorinated high-density polyethylene (HDPE) containers demonstrate leaching of PFAS into stored liquids, with temperature and storage time acting as important modifiers [51]. Beyond post-production influences, environmental and agricultural factors may also contribute to variability in PFAS levels in dairy. Seasonal dynamics in livestock exposure have been documented, where changes in grazing behavior, feed composition, and water sources across the year impact PFAS uptake and body burden in animals [52]. Within agri-food systems, dairy cattle exposed through contaminated feed or water have shown PFAS accumulation and secretion into milk, with exposure varying according to environmental conditions and management practices [53]. In parallel, studies of raw milk composition confirm strong seasonal variability in fat and protein profiles, which may further influence the partitioning or binding of PFAS in dairy products [54].

Our study design controlled for several factors, such as using polypropylene food grade containers and consistent cold-chain conditions during storage and transport, thereby reducing the likelihood of artificial contamination during handling. However, we did not evaluate seasonal variation in raw milk composition, feed and water contamination, or differences in production equipment and storage practices across governorates. These variables may explain the regional differences observed in PFAS levels, such as the higher PFDS concentrations in some areas, and should be investigated in future longitudinal and supply-chain-based studies. Addressing these aspects would not only strengthen causal interpretation but also provide essential evidence for policymakers to target interventions across different stages of dairy production.

While acute health risks from current PFAS levels in Lebanese yogurt appear low, the observed spatial variation, particularly elevated contamination in Bekaa, North Lebanon, and Beirut-Hermel (BH), calls for proactive food safety and environmental interventions. As yogurt is a widely consumed staple in the Lebanese diet, even low-level contamination can contribute to chronic PFAS exposure. Routine surveillance of dairy products, including yogurt, should be prioritized in regions identified as hotspots. In parallel, biomonitoring studies, especially among sensitive groups such as infants, children, and breastfeeding women, are needed to evaluate internal exposure levels and verify potential health impacts. Lebanon currently lacks food-specific regulatory limits for PFAS. Therefore, establishing national standards for PFAS residues in dairy products, aligned with international guidelines from the European Food Safety Authority (EFSA) and the U.S. Environmental Protection Agency (USEPA), is strongly recommended [1,2]. Furthermore, strategies such as strengthening farm-level water quality controls, restricting PFAS use in agricultural packaging and feed systems, and educating producers and consumers on PFAS risks should be adopted to reduce exposure across the food chain.

Finally, it should be emphasized that references to “potential health risks” in this study pertain specifically to long-term, cumulative exposure rather than acute toxicity. Repeated consumption of widely eaten foods such as yogurt may contribute to chronic PFAS exposure, particularly in sensitive populations including infants, children, and pregnant women. These findings underscore the importance of routine food surveillance, targeted biomonitoring, and the establishment of regulatory measures aligned with international guidelines to mitigate exposure and protect public health.

### 4.7. Limitations

This study is subject to certain limitations, including its restriction to a single season (spring), the lack of investigation into potential production-related factors such as raw milk, processing equipment, and storage materials that may influence PFAS levels, and the limited representativeness of the dataset with respect to industrial dairy production and intra-regional variability. Future research should address these aspects to provide a more comprehensive understanding of contamination pathways and population exposure.

While food-grade polypropylene is less likely to leach PFAS compared to fluoropolymer-coated materials, the type of container remains a potential factor influencing contamination and thus merits consideration in future studies. Concerning risk assessment calculations, we acknowledge that children represent a particularly vulnerable group due to higher intake-to-body weight ratios and developmental sensitivity, and future studies should prioritize exposure assessment in this subgroup once nationally representative data become available

## 5. Conclusions

The study found significant geographic variability in PFAS concentrations across Lebanese governorates, with the Bekaa region exhibiting the highest contamination levels for multiple PFAS compounds, suggesting it as a potential environmental hotspot. Elevated concentrations were also observed in North Lebanon and Beirut-Hermel, while regions like Jbeil showed consistently lower PFAS levels. Health risk assessment indicated that, although estimated daily intakes and risk quotients for all measured PFAS were below established safety thresholds, the cumulative hazard index averaged 0.71, approaching the level warranting caution. This suggests that while acute health risks may be limited, the possibility of chronic effects due to cumulative exposure, especially in high-burden regions, cannot be overlooked. Therefore, the findings underscore the urgency of continuous environmental monitoring, public health surveillance, and the development of regulatory frameworks to manage PFAS pollution effectively in Lebanon.

## Figures and Tables

**Table 1:** The PFAS compounds and their RfD/TDI values (in ng/kg bw/day).

PFAS Compound	RfD/TDI Value (ng/kg bw/day)	Source/Reference	Notes/Comments
PFOS	0.63	EFSA (2020)—Group TWI [1]	EFSA TWI of 4.4 ng/kg bw/week for sum of PFOS, PFOA, PFNA, PFHxS; divided equally = 0.63 ng/kg bw/day per compound
PFOA	0.63	EFSA (2020)—Group TWI [1]	Same group TWI as PFOS
PFNA	0.63	EFSA (2020)—Group TWI [1]	Part of group TWI
PFHxS	0.63	EFSA (2020)—Group TWI [1]	Included in EFSA group TWI
PFHpA	0.63 (surrogate)	US EPA and EFSA surrogate approach [25]	No specific RfD; surrogate uses PFOA’s value
PFBA	10,000	US EPA (2022) PPRTV [26]	US EPA Provisional Peer-Reviewed Toxicity Value
PFBS	3000	US EPA (2022) Health Advisory [3]	Based on EPA Health Advisory
PFPeA	0.63 (surrogate)	US EPA/EFSA surrogate approach [25]	No official RfD; surrogate with PFOA RfD
PFDA	0.3	ATSDR (2021) MRL [27]	Minimal Risk Level for chronic oral exposure
PFUnDA	0.3	ATSDR (2021) MRL [27]	Surrogate based on PFDA
PFDoDA	0.3 (surrogate)	EFSA/ATSDR surrogate approach [27]	No official RfD; considered similar to PFUnDA
GenX (HFPO-DA)	3	US EPA (2022) Health Advisory [3]	EPA final health advisory value
ADONA	No established RfD	Limited data, under review [27]	Data insufficient for quantitative RfD

**Table 2:** Mean Concentrations (ng/kg), 95% Confidence Intervals, and *p*-Values of Perfluoroalkyl Carboxylic Acids (PFAs) in yogurt Samples from Lebanese Regions.

		Akkar	Baalbeck-Hermel	Batroun	Bekaa	Chouf	Jbeil	Kesserwan	Metn	Nabatiyeh	North Area	South Lebanon
PFHpA	*p* value	<0.001	<0.001	<0.001	<0.001	<0.001	<0.001	<0.001	<0.001	<0.001	<0.001	<0.001
	Mean	29.69	30.00	27.51	32.77	26.51	24.40	26.35	26.64	28.32	31.69	28.55
	95% CI	25.11–34.27	25.36–34.65	21.31–33.70	27.67–37.86	20.51–32.51	17.42–31.38	20.42–32.28	20.60–32.68	21.88–34.75	26.65–36.72	22.01–35.09
PFOA	*p* value	<0.001	<0.001	<0.001	<0.001	<0.001	<0.001	<0.001	<0.001	<0.001	<0.001	<0.001
	Mean	146.65	150.40	129.55	153.60	126.72	107.64	120.50	113.18	127.98	135.30	136.10
	95% CI	141.6–151.7	145.4–155.4	99.80–159.3	147.8–159.4	97.84–155.6	76.68–138.6	92.90–148.1	80.55–145.8	98.65–157.3	113.7–156.9	114.4–157.8
PFNA	*p* value	0.021	0.003	0.02142	0.886	0.016	<0.001	0.001	0.003	0.219	0.677	0.043
	Mean	4.21	4.83	3.76	4.35	3.23	2.43	3.72	3.18	3.87	4.31	3.54
	95% CI	3.891–4.522	4.576–5.091	3.148–4.372	3.666–5.041	2.318–4.149	1.679–3.188	3.355–4.085	2.449–3.911	2.996–4.751	3.876–4.751	2.713–4.367
PFHxS	*p* value	<0.001	<0.001	<0.001	<0.001	<0.001	<0.001	<0.001	<0.001	<0.001	<0.001	<0.001
	Mean	75.87	77.20	70.42	79.40	71.95	55.86	61.04	62.57	66.91	67.98	68.58
	95% CI	72.69–79.05	74.05–80.35	59.03–81.81	76.67–82.13	60.52–83.38	39.62–72.10	43.91–78.17	48.07–77.07	51.76–82.05	52.41–83.54	52.77–84.38
PFOS	*p* value	<0.001	<0.001	<0.001	<0.001	<0.001	<0.001	<0.001	<0.001	<0.001	<0.001	<0.001
	Mean	151.35	162.60	150.05	153.65	149.60	153.95	147.85	156.95	160.95	174.30	159.90
	95% CI	117.2–185.5	137.3–187.9	116.1–184.0	119.0–188.3	115.8–183.4	129.8–178.1	114.3–181.4	132.3–181.6	135.6–186.3	169.6–179.0	134.8–185.0
PFHxA	*p* value	<0.001	<0.001	<0.001	<0.001	<0.001	<0.001	<0.001	<0.001	<0.001	<0.001	<0.001
	Mean	31.68	37.33	31.75	38.94	25.80	28.11	29.03	26.74	30.55	33.26	30.90
	95% CI	30.89–32.47	36.00–38.65	30.87–32.63	37.23–40.64	18.29–33.30	23.74–32.48	24.44–33.61	20.63–32.85	25.44–35.65	25.70–40.82	23.64–38.15
PFBA	*p* value	<0.001	<0.001	<0.001	<0.001	<0.001	<0.001	<0.001	<0.001	<0.001	<0.001	<0.001
	Mean	137.95	157.65	128.49	156.60	140.60	126.66	137.05	126.36	130.85	153.95	141.40
	95% CI	116.0–159.9	152.2–163.1	99.07–157.9	148.6–164.6	117.9–163.3	96.51–156.8	115.5–158.6	97.11–155.6	100.8–160.9	145.4–162.5	118.9–163.9
PFPeA	*p* value	<0.001	<0.001	<0.001	<0.001	<0.001	<0.001	<0.001	<0.001	<0.001	<0.001	<0.001
	Mean	68.96	78.47	59.00	77.14	66.58	62.71	72.80	57.60	78.13	66.58	67.64
	95% CI	57.94–79.97	75.14–81.79	41.87–76.12	73.36–80.91	51.08–82.07	48.08–77.33	69.32–76.28	40.85–74.34	74.33–81.93	51.10–82.05	51.96–83.32

**Table 3:** One-Way ANOVA Test for Comparing the Means of Each Compound in the various Regions.

	F	*p* Value
PFHpA	0.840	0.591
PFOA	1.722	0.080
PFNA	4.754	<0.001
PFHxS	1.470	0.156
PFOS	0.335	0.970
PFHxA	3.047	0.001
PFBA	1.229	0.277
PFPeA	1.609	0.109

F (or F statistic) is a ratio of two variances used to test for statistical significance of the differences between the means of multiple samples. F is usually compared to a critical value from the F-distribution (Fisher–Snedecor distribution) to obtain a corresponding *p*-value, which is the level of significance of the results. If *p* < 0.05, then the results are significant which means the samples have significantly different means. Otherwise, if *p* > 0.05, then the results are not significant (NS) which means the samples’ means are not significantly different from each other.

**Table 4:** Calculation of EDI, RQ and HI of PFAs in Lebanese yogurt across Lebanese governorates (ng/kg).

	Nabatieh	South	Chouf	Metn	Keserwan	Jbeil	Batroun	North Lebanon	Akkar	Bekaa	BH	Average
PFHpA												
Concentration	32.62	32.89	30.54	30.69	30.35	30.42	31.69	33.93	31.78	35.08	32.13	32.01
EDI	0.03	0.03	0.03	0.03	0.03	0.03	0.03	0.03	0.03	0.03	0.03	0.03
RQ	0.05	0.05	0.05	0.05	0.05	0.05	0.05	0.05	0.05	0.05	0.05	0.05
PFOA												
Concentration	147.61	145.78	146.15	141.42	139	134.50	149.46	144.93	146.67	153.60	150.40	145.42
EDI	0.14	0.14	0.143	0.14	0.14	0.13	0.15	0.14	0.14	0.15	0.15	0.14
RQ	0.23	0.23	0.23	0.22	0.22	0.21	0.23	0.22	0.23	0.24	0.23	0.22
PFNA												
Concentration	4.42	4.04	3.97	3.62	3.72	3.21	4.01	4.31	4.21	4.64	4.83	
EDI	0.004	0.004	0.004	0.003	0.004	0.003	0.004	0.004	0.004	0.004	0.005	0.004
RQ	0.007	0.006	0.006	0.006	0.0067	0.005	0.006	0.007	0.006	0.007	0.007	0.006
PFDA												
Concentration	ND	ND	ND	ND	ND	ND	ND	ND	ND	ND	ND	
EDI	0	0	0	0	0	0	0	0	0	0	0	0
RQ	0	0	0	0	0	0	0	0	0	0	0	0
PFHxS												
Concentration	77.15	79.08	77.07	72.15	70.38	69.75	75.43	78.38	75.87	79.40	77.20	
EDI	0.07	0.08	0.07	0.07	0.07	0.07	0.07	0.08	0.07	0.08	0.07	0.07
RQ	0.12	0.12	0.12	0.11	0.11	0.11	0.12	0.12	0.12	0.12	0.12	0.12
PFOS												
Concentration	172.43	171.28	172.54	168.14	170.54	164.93	173.08	174.23	174.61	177.23	174.21	
EDI	0.17	0.168	0.17	0.16	0.17	0.16	0.17	0.17	0.17	0.17	0.17	0.17
RQ	0.27	0.27	0.27	0.26	0.26	0.26	0.27	0.27	0.275	0.27	0.27	0.27
PFHxA												
Concentration	32.714	35.60	32.17	30.81	31.08	30.09	31.75	38.33	31.68	38.93	37.33	
EDI	0.03	0.03	0.03	0.03	0.03	0.03	0.03	0.04	0.03	0.04	0.04	0.03
RQ	6.40 × 10^−5^	6.97× 10^−5^	6.30 × 10^−5^	6.03 × 10^−5^	6.08 × 10^−5^	5.89 × 10^−5^	6.22 × 10^−5^	7.50 × 10^−5^	6.20 × 10^−5^	7.62 × 10^−5^	7.31 × 10^−5^	11. 66 × 10^−5^
PFBA												
Concentration	150.92	151.50	150.64	145.77	146.86	146.08	148.23	153.93	147.78	156.60	157.676	
EDI	0.15	0.15	0.15	0.141	0.14	0.14	0.14	0.15	0.15	0.15	0.15	0.15
RQ	1.48 × 10^−5^	1.48 × 10^−5^	1.47 × 10^−5^	1.43 × 10^−5^	1.44 × 10^−5^	1.43 × 10^−5^	1.45 × 10^−5^	1.51 × 10^−5^	1.45 × 10^−5^	1.53 × 10^−5^	1.54× 10^−5^	1.47 × 10^−5^
PFBS												
Concentration	ND	ND	ND	ND	ND	ND	ND	ND	ND	ND	ND	
EDI	0	0	0	0	0	0	0	0	0	0	0	0
RQ	0	0	0	0	0	0	0	0	0	0	0	0
PFPeA												
Concentration	78.13	78	76.77	71.92	72.80	72.31	73.67	76.77	73.86	77.13	78.47	
EDI	0.08	0.076	0.075	0.07	0.07	0.07	0.07	0.07	0.07	0.07	0.08	0.07
RQ	0.12	0.12	0.12	0.11	0.11	0.11	0.11	0.12	0.11	0.12	0.12	
Hazard Index	0.68	0.68	0.67	0.65	0.65	0.64	0.67	0.69	0.67	0.71	0.70	0.67

## Data Availability

The data are contained within the article. Additional data are available upon request from the corresponding authors.

## Supplementary Materials

The following supporting information can be downloaded at: https://www.mdpi.com/article/10.3390/foods14203472/s1, Table S1: Central GPS coordinates of yogurt sampling locations across Lebanese governorates.
