# Supplementary material for: Occurrence and Health Risk Assessment of Per- and Polyfluoroalkyl Substances in Yogurt Across Lebanese Governorates
Source: Foods. 2025 Oct 11;14(20):3472. doi: 10.3390/foods14203472 (PMC12564224; doi:10.3390/foods14203472)
Supplement: Supplementary file 1 [file foods-14-03472-s001.zip › foods-3873505-supplementary.pdf]

Supplementary Material

Table S1. Central GPS coordinates of yogurt sampling locations across Lebanese governorates.

**Governorate**	**Sampling Location (City/Town)**	**Latitude (°N)**	**Longitude (°E)**
Nabatieh (Na)	Nabatieh	33.3773	35.4836
South Lebanon (S)	Saida (Sidon)	33.5606	35.3756
Chouf (C)	Beiteddine (Chouf)	33.6942	35.5769
Metn (M)	Sin el Fil (Metn)	33.8683	35.5426
Keserwan (K)	Jounieh	33.9800	35.6400
Jbeil (J)	Byblos (Jbeil)	34.1203	35.6481
Batroun (B)	Batroun	34.2558	35.6581
North Lebanon (No)	Tripoli	34.4367	35.8497
Akkar (A)	Halba	34.5420	36.0798
Bekaa (Be)	Zahle	33.8467	35.9020
Baalbeck-Hermel (BH)	Baalbeck	34.0058	36.2181
